# Clinical and sociodemographic correlates of body mass index in a large multicenter obsessive–compulsive disorder sample

**DOI:** 10.1007/s40519-026-01869-x

**Published:** 2026-06-18

**Authors:** Samuel Frota Cunha, Jônatas Magalhães Santos, Acácio Moreira-Neto, Roseli Gedanke Shavitt, Ygor Arzeno Ferrão, Maria Conceição do Rosário, Maria Alice Simões de Mathis, Marcos Vinícius Sousa de Oliveira, Leonardo F. Fontenelle, Euripedes Constantino Miguel, Marcelo Camargo Batistuzzo, Marcelo Queiroz Hoexter

**Affiliations:** 1 Department of Psychiatry, University of São Paulo, Rua Dr. Ovídio Pires de Campos, 785, São Paulo, Brazil; 2 Department of Psychiatry, Federal University of Health Sciences of Porto Alegre, Porto Alegre, Brazil; 3 Child and Adolescent Psychiatry Unit, Department of Psychiatry, Federal University of São Paulo, São Paulo, Brazil; 4 Institute of Psychiatry, Federal University of Rio de Janeiro, Rio de Janeiro, Brazil; 5 D’Or Institute for Research and Education, Rio de Janeiro, Brazil; 6 Department of Psychiatry, Monash University, Melbourne, Australia; 7 Department of Methods and Techniques, Faculty of Human and Health Sciences, Pontifical Catholic University of São Paulo, São Paulo, Brazil; 8 Hospital Israelita Albert Einstein, São Paulo, SP Brazil

**Keywords:** Obsessive–compulsive disorder, Body mass index, Body dysmorphic disorder, Demography

## Abstract

**Purpose:**

This multicenter study aims to investigate associations between body mass index (BMI) and sociodemographic and clinical characteristics in a clinical sample of individuals with obsessive–compulsive disorder (OCD).

**Methods:**

Data were analyzed from 947 adults diagnosed with OCD, recruited through the Brazilian Research Consortium on Obsessive–Compulsive Spectrum Disorders. BMI was calculated from self-reported weight and height and categorized according to WHO definitions. Sociodemographic and clinical data were obtained using standardized instruments. Associations between BMI and sociodemographic/clinical variables were examined using linear regression models.

**Results:**

The mean BMI in the sample was 24.55 kg/m^2^ (SD = 4.65), with 5.5% of participants classified as underweight, 58% as normal weight, 25% as overweight, and 11.5% as obese. In the multivariate linear regression model, higher BMI was significantly associated with older age (*β* = 0.09, *p* < 0.001), current psychiatric treatment (*β* = 1.4, *p* < 0.001), and binge eating disorder (*β* = 4.4, *p* < 0.001). Lower BMI was significantly associated with female sex (*β* = – 1.6, *p* < 0.001) and body dysmorphic disorder (*β* = – 1.2, *p* = 0.005). No significant associations were found with educational level, OCD severity, comorbid depression, anxiety, bulimia, or anorexia.

**Conclusion:**

Although BMI was unrelated to OCD severity, it was associated with demographic, clinical, and treatment-related variables, underscoring the need for evaluation beyond symptom-based assessment.

*Level of evidence:* III, as it is based on an observational analytic design using a large cross-sectional multicenter sample.

## Introduction

Obsessive–compulsive disorder (OCD) is a psychiatric condition characterized by intrusive thoughts, fears, preoccupations, and/or impulses (obsessions) and/or repetitive behaviors (compulsions) [1]. The symptoms typically begin in the first decade of life, with an earlier onset in men [2]. It is usually diagnosed in late adolescence or early adulthood, most commonly between the ages of 18 and 20, and tends to have a chronic course [2, 3]. The prevalence of OCD is approximately 2.5% worldwide [4] and around 90% of adults with OCD present with psychiatric comorbidities, most commonly mood and anxiety disorders, followed by substance use and impulse-control disorders [2]. Moreover, patients with OCD present with elevated rates of non-psychiatric comorbidities compared to the general population and experience a reduced quality of life, similar to that observed in patients with schizophrenia [4, 5].

Several psychiatric disorders have been associated with increased body mass index (BMI) and cardiovascular risk, either due to the direct impact of the disorder on physical health or as a consequence of psychotropic medications adversely affecting metabolic profiles [5, 6]. This association increases morbidity, mortality, and functional impairment [7]. OCD is no exception to most of these patterns [8]. A retrospective Swedish cohort study concluded that individuals with OCD had a 45% higher risk of developing metabolic disorders, such as obesity, and/or cardiovascular complications compared to the general population [8]. Although increased cardiovascular risk is typically associated with higher BMI [8], studies investigating the relationship between BMI and OCD have yielded mixed results.

Contrary to expectations regarding BMI in OCD, emerging evidence suggests that this relationship may differ, with OCD patients potentially presenting lower BMI levels compared to other psychiatric and general populations [5, 9, 10]. A Mendelian randomization framework study found an inverse relationship between BMI and risk of OCD [5]. Also, a recent genome-wide association meta-analysis demonstrated a negative genetic correlation between OCD and BMI. In other words, genes associated with an increased risk for OCD were also associated with risk for lower BMI [11]. Similar findings were observed in a Dutch study, which reported that patients with “pure” OCD, those without depressive comorbidity, had lower BMIs compared with individuals with anxiety, depression, or both disorders [9]. Consistently, Subramaniam et al. [10] also found that being underweight was associated with both a lifetime and 12-month OCD prevalence [10].

The current understanding of mental health increasingly emphasizes health parameters beyond core psychiatric symptoms, underscoring the need for a more integrative approach to OCD management [12]. It is well established that patients with OCD experience poorer quality of life, unhealthier diets, including increased consumption of high-calorie foods, and, as a result, higher rates of cardiovascular morbidity and mortality [8, 13, 14]. Therefore, factors such as BMI should be considered when evaluating the overall health of these individuals.

BMI variation in OCD may be viewed as a multidimensional concept involving pharmacological, neurobiological, and behavioral pathways. Psychotropic medications, especially antipsychotic augmentation and some antidepressants, are associated with weight gain [15, 16]. Neurobiologically, changes in reward and anhedonia circuits may reduce the pleasure derived from food, possibly lowering caloric intake [17]. From a behavioral and psychopathological standpoint, OCD symptoms may overlap with disordered eating patterns, including dietary restriction, body dissatisfaction, and drive for thinness [18]. Rigid, rule-governed eating behaviors and orthorexic tendencies have been observed in OCD populations [19]. Symptom exacerbation during mealtimes may further disrupt eating patterns [20]. Together, these mechanisms may contribute to BMI variability in OCD, but their relative contributions are still unclear.

In summary, the available literature provides conflicting results in terms of BMI in OCD [5, 8–11, 21]. In light of these inconsistencies, and considering that BMI shows a relationship with multiple established factors in psychiatric samples**,** we expected BMI to be primarily associated with sociodemographic and clinical factors. The association between BMI and OCD symptom severity was also examined. Furthermore, most available data pertain to populations in developed countries, highlighting a lack of data from low- and middle-income countries. Therefore, this study investigates a Brazilian population diagnosed with OCD, from the Brazilian Research Consortium on Obsessive–Compulsive Spectrum Disorders (C-TOC) [22] and aims to: (1) assess the distribution of underweight, normal weight, overweight, and obesity in these patients; and (2) identify which sociodemographic and clinical characteristics are associated with BMI.

## Methods

### Study design and sample

This is an observational, cross-sectional, and analytical study. Data were analyzed from a clinical sample of 1001 OCD participants from seven OCD centers across three distinct regions of Brazil (south, southeast, and northeast). Participants were recruited between August 2003 and March 2009, as part of the nationwide C-TOC [22]. Inclusion criteria consisted of a primary psychiatric diagnosis of OCD according to the Diagnostic and Statistical Manual of Mental Disorders, Fourth Edition (DSM-IV) [23], confirmed with an interview with the Structured Clinical Interview for DSM-IV Axis I Disorders (SCID-I) [24]. Exclusion criteria were: the diagnosis of schizophrenia and/or intellectual disability, or any condition that could interfere with the ability to understand the research questions. For the present analyses, participants younger than 18 years (*n* = 46) and those with missing data (*n* = 8) were excluded, yielding a final sample of 947 individuals. Data collection was performed by trained psychiatrists and/or clinical psychologists using a comprehensive set of standardized instruments to assess sociodemographic and clinical characteristics, described below.

### Assessments

The study protocol included the SCID-I and additional modules for tic and impulse-control disorders [24], the Yale-Brown Obsessive–Compulsive Scale (Y-BOCS) [25], the Beck Depression Inventory (BDI) [26], and the Beck Anxiety Inventory (BAI) [27]. BMI was calculated from self-reported weight and height (kg/m^2^). All participants or their legal guardians provided written informed consent before enrollment in the study. The procedures were conducted in accordance with the ethical standards of the Research Ethics Committee and the 1964 Helsinki Declaration and its later amendments.

### Data analyses

The primary outcome of this study was BMI, calculated as weight (kg) divided by height squared (m^2^). BMI was treated in two ways: (i) categorical, following the World Health Organization thresholds for: underweight (< 18.5 kg/m^2^), normal weight (18.5–24.9 kg/m^2^), overweight (25–29.9 kg/m^2^), and obesity (≥ 30 kg/m^2^); and (ii) as a continuous variable, for all inferential statistics [28]. Prior to the statistical analyses, extreme outliers in height and weight values were identified and excluded from the dataset. Outliers were identified beforehand based on implausible anthropometric measurements. Specifically, height values < 1.30 m (*n* = 3) and weight values < 30 kg (*n* = 5) were considered likely data-entry errors and treated as missing. Regarding the sociodemographic variables, sex, age, educational level (categorized as completed high school or higher vs. less than high school), socioeconomic class (classified as A/B vs. C/D/E according to the criteria of the Brazilian Association of Research Companies—ABEP), and self-reported ethnicity (White vs. non-White) were included. Among the clinical variables, the following were considered: severity of the obsessive–compulsive symptoms, as measured by the Y-BOCS; presence of psychiatric comorbidities, assessed using the SCID-I, including mood disorders, anxiety disorders, substance use disorders, eating disorders, and body dysmorphic disorder (BDD), depressive symptoms, measured by the BDI; anxiety symptoms, assessed by the BAI; and current psychiatric treatment (yes/no) indicated whether participants were receiving psychiatric care with ongoing pharmacological treatment at the time of assessment. Psychotherapy was not captured by this variable.

Statistical analyses were conducted using the R software (version 4.5.0). The normality of BMI distribution was assessed with the Shapiro–Wilk test. As BMI was not normally distributed, non-parametric tests (Kruskal–Wallis test and Dunn’s post hoc test) were used to compare more than two groups. For comparisons involving exactly two groups, we employed the Welch’s t-test, which does not assume equal variances and remains robust to moderate deviations from normality, particularly in large samples. In addition, effect sizes (Cohen’s d) were calculated for all binary comparisons to quantify the magnitude of BMI differences between groups. Spearman’s correlations were used to assess associations between BMI and continuous clinical variables. To prevent type I error inflation from multiple pairwise comparisons, p-values from bivariate analyses were adjusted using the false discovery rate (FDR) correction (Benjamini–Hochberg method). Multivariate linear regression models were used to analyze the relationship between continuous BMI and the selected sociodemographic and clinical variables. Variables were chosen for inclusion in the final model based on their clinical and epidemiological relevance to BMI and body composition, as consistently reported in the literature. P-values from the regression models were not adjusted for multiple comparisons, as inference was based on a single multivariable model in which all predictors were estimated simultaneously (model-based inference). Due to the very low prevalence, some comorbidities were not included in the regression models because the small number of cases did not allow reliable effect estimates.

Although BMI was not normally distributed, linear regression was considered appropriate as it assumes normality of residuals rather than the outcome. Residual diagnostics were conducted, and considering the substantial sample size, the central limit theorem underpins the asymptotic validity of the estimates. Model assumptions were further evaluated using variance inflation factors (VIF). Heteroscedasticity was assessed using the Breusch–Pagan test and addressed using HC3 robust standard errors. Given the multicenter design, a mixed-effects model with a random intercept for center was also tested and compared with the ordinary linear model using a likelihood ratio test and the intraclass correlation coefficient (ICC), and information criteria (Akaike information criterion [AIC] and Bayesian information criterion [BIC]). To assess the robustness of the findings to deviations from normality and to extreme values, a sensitivity analysis was conducted using median-quantile regression (*τ* = 0.50) with bootstrap standard errors (1,000 replications), including the same set of predictors as in the primary model. A significance level of *p* < 0.05 was adopted for all analyses.

## Results

### Sociodemographic and clinical characteristics of OCD

The study sample consisted of 947 participants, with a predominance of females (58%). Most were self-identified as White (83%), followed by Black or mixed individuals (16%). The mean age of the sample was 33 years. Higher education was predominant in the sample, with 34% of participants holding a postgraduate degree. Additionally, the majority of the sample (56%) belonged to higher socioeconomic classes (A and B). Comorbidity data showed that 6.7% of the sample had binge eating disorder (BED), 1.8% bulimia nervosa, 1.3% anorexia nervosa, 11% BDD, 6.2% substance use disorder, 35% major depressive disorder, and 63.8% anxiety disorder.

### BMI and OCD

**Figure.1:**
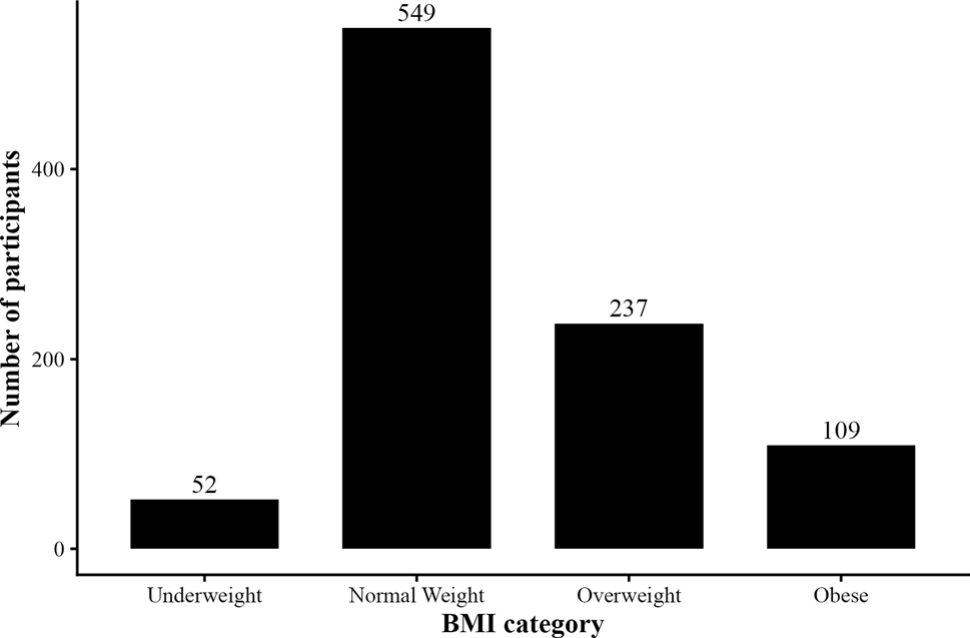
Body mass index distribution of the sample according to the WHO criteria

The mean BMI of the sample was 24.55 kg/m^2^ (SD = 4.65). According to the WHO criteria, the BMI distribution among participants was as follows: 52 (5.5%) were classified as underweight; 549 (58%) as normal weight; 237 (25%) as overweight; and 109 (11.5%) as obese (Fig. 1; Table 1).

**Table 1:** Descriptive characteristics of the sample (N = 947)

Characteristics	Overall N = 947	BMI categories*				p-value
		Underweight N = 52	Normal weight N = 549	Overweight N = 237	Obese N = 109	
**Sociodemographic characteristics**						
Age (years)^1^	33.0 (25.0–45.0)	26.5 (22.0–36.5)	31.0 (25.0–42.0)	38.0 (29.0–49.0)	39.0 (31.0–48.0)	** < 0.001**
Sex^2^						** < 0.001**
Male	398 (42%)	10 (19%)	210 (38%)	129 (54%)	49 (45%)	
Female	549 (58%)	42 (81%)	339 (62%)	108 (46%)	60 (55%)	
Ethnicity^2^						0.331
Caucasian	786 (83%)	42 (81%)	464 (85%)	196 (83%)	84 (77%)	
Black or mixed	147 (16%)	9 (17%)	79 (14%)	35 (15%)	24 (22%)	
Asian and others	14 (1.5%)	1 (1.9%)	6 (1.1%)	6 (2.5%)	1 (0.9%)	
Education level^2^						0.218
< High school	160 (17%)	8 (15%)	83 (15%)	43 (18%)	26 (24%)	
Completed high school	280 (30%)	19 (37%)	163 (30%)	67 (28%)	31 (28%)	
Incomplete higher education	185 (20%)	14 (27%)	113 (21%)	40 (17%)	18 (17%)	
Post-graduation	321 (34%)	11 (21%)	189 (34%)	87 (37%)	34 (31%)	
Socioeconomic status^2^						0.145
Classes A/B (higher)	526 (56%)	21 (40%)	306 (56%)	136 (57%)	63 (58%)	
Classes C/D/E (lower)	421 (44%)	31 (60%)	243 (44%)	101 (43%)	46 (42%)	
Collection site^2^						0.285
São Paulo/Rio de Janeiro	557 (59%)	31 (60%)	316 (58%)	143 (60%)	67 (61%)	
Bahia/Pernambuco	165 (17%)	14 (27%)	96 (17%)	41 (17%)	14 (13%)	
Rio Grande do Sul	225 (24%)	7 (13%)	137 (25%)	53 (22%)	28 (26%)	
**Clinical variables**^*2*^						
Current psychiatric treatment	724 (76%)	31 (60%)	400 (73%)	199 (84%)	94 (86%)	** < 0.001**
Binge eating disorder	63 (6.7%)	0 (0%)	23 (4.2%)	18 (7.6%)	22 (20%)	** < 0.001**
Body dysmorphic disorder	106 (11%)	9 (17%)	66 (12%)	27 (11%)	4 (3.7%)	**0.036**
Bulimia nervosa	17 (1.8%)	0 (0%)	11 (2.0%)	4 (1.7%)	2 (1.8%)	0.974
Anorexia nervosa	12 (1.3%)	2 (3.8%)	8 (1.5%)	0 (0%)	2 (1.8%)	**0.047**
Major depressive disorder	332 (35%)	18 (35%)	192 (35%)	78 (33%)	44 (40%)	0.607
Anxiety disorder	603 (64%)	35 (67%)	348 (63%)	152 (64%)	68 (62%)	0.937
Substance use disorder	59 (6.2%)	2 (3.8%)	34 (6.2%)	15 (6.3%)	8 (7.3%)	0.895
**Psychometric scales**^*1*^						
Y-BOCS	26.0 (21.0–31.0)	27.0 (24.0–31.0)	26.0 (22.0–31.0)	26.0 (20.0–31.0)	25.0 (20.0–29.0)	0.133
Beck anxiety inventory	14.0 (7.0–24.0)	14.5 (7.0–24.0)	14.0 (7.0–24.0)	14.0 (7.0–25.0)	15.0 (7.0–24.0)	> 0.999
Beck depression inventory	16.0 (8.0–24.0)	15.0 (8.0–24.5)	15.0 (8.0–23.0)	16.0 (6.0–22.0)	18.0 (9.0–25.0)	0.450

P-values reported are descriptive and unadjusted, intended to characterize the distribution of sample characteristics across BMI categories

^*^BMI categories were defined according to WHO criteria: underweight (< 18.5 kg/m^2^), normal weight (18.5–24.9 kg/m^2^), overweight (25.0–29.9 kg/m^2^), and obesity (≥ 30.0 kg/m^2^)

^1^Continuous variables are presented as median (IQR) and compared using the Kruskal–Wallis rank-sum test

^2^Categorical variables are presented as n (%) and compared using Pearson’s Chi-squared test; Fisher’s exact test was used when expected cell counts were low

### Associations between BMI and sociodemographic/clinical variables

A positive and statistically significant association was seen between age and BMI (*ρ* = 0.28, *p* < 0.001), reflecting a small-to-moderate effect. Men had a significantly higher mean BMI compared to women (*n* = 398 [25.35 kg/m^2^] vs. *n* = 549 [23.98 kg/m^2^]; *p* < 0.001; *t* = 4.570; *d* = 0.30), corresponding to a small effect size. In terms of socioeconomic status, no significant differences in mean BMI were found between participants with higher (A and B) versus lower (C, D, and E) socioeconomic status (*p* = 0.21; *d* = 0.11, negligible).

No significant correlation was observed between BMI and overall OCD severity (*ρ* = − 0.07, *p* = 0.073). Patients currently undergoing psychiatric treatment had higher BMI compared to those not in treatment (mean BMI = 24.91 vs. 23.39; *d* = 0.33; *p* < 0.001), representing a small effect size. There was no significant correlation between BMI and age at OCD symptom onset (*ρ* = 0.03, *p* = 0.58; negligible effect). When examining data on eating disorders, bivariate analyses revealed no significant associations between BMI and the presence of bulimia (*p* = 0.71; *d* = 0.11) or anorexia (*p* = 0.43; *d* = 0.43). However, a statistically significant association was found for higher BMI among individuals with BED (mean = 28.59 vs. 24.27; *p* < 0.001, *d* = 0.95), corresponding to a large effect size. Additionally, individuals with BDD had a significantly lower BMI compared to those without the disorder (mean = 23.40 vs. 24.70; *p* = 0.006, *d* = 0.28), reflecting a small effect size.

### Multiple linear regression model

The severity of obsessive–compulsive symptoms, measured by the Y-BOCS total score, was not related to BMI in the multivariable linear regression model (*β* = – 0.032, *p* = 0.093; Table 2). Likewise, educational level showed no significant association with BMI. Female sex, however, was linked to a lower BMI (*β* = – 1.6, *p* < 0.001), a modest effect. As expected, the presence of BED was positively associated with higher BMI (*β* = 4.4, *p* < 0.001), representing the largest effect in the model. Current psychiatric treatment was also associated with higher BMI (*β* = 1.4, *p* < 0.001), a small but clinically relevant difference. By contrast, BDD was negatively associated with BMI (*β* = – 1.24, *p* = 0.005), reflecting a small effect in the opposite direction. Finally, older age remained significantly associated with higher BMI, with an average increase of 0.09 kg/m^2^ per year (*β* = 0.09, *p* < 0.001), a small effect. We found minimal clustering by center, with a low intraclass correlation coefficient (ICC ≈ 0.007). The mixed-effects model showed no meaningful improvement over the ordinary linear model (ΔAIC < 2; LRT *p* = 0.206). Accordingly, the ordinary linear model was retained as the final model due to its comparable performance and greater parsimony.

**Table 2:** Multiple linear regression model

Predictor	Beta (B)	95% CI	p-value
Y-BOCS (total)	– 0.03	– 0.07, 0.01	0.093
Education level			
From illiterate to uncompleted 2º level	–	–	
Completed 2º level	– 0.60	– 1.4, 0.25	0.165
Uncompleted 3º level	– 0.38	– 1.3, 0.56	0.429
Post-graduation	– 0.57	– 1.4, 0.25	0.175
Sex			
Male	–	–	
Female	– 1.6	– 2.2, – 1.0	** < 0.001**
Binge eating disorder			
No	–	–	** < 0.001**
Yes	4.4	3.3, 5.5	
Current psychiatric treatment			
No	–	–	** < 0.001**
Yes	1.4	0.73, 2.0	
Body dysmorphic disorder			
No	–	–	**0.005**
Yes	– 1.2	– 2.1, – 0.37	
Age (years)	0.09	0.07, 0.11	** < 0.001**

General model: adjusted R^2^ = 0.157, F(9, 936) = 20.51, p < 0.001. Reference categories correspond to the first level of each categorical variable and are indicated by “–” in the table: male (sex); from illiterate to uncompleted high school (education level); absence of BED; no current psychiatric treatment; and absence of BDD

CI: confidence interval

### Sensitivity and secondary analysis

Median quantile regression (*τ* = 0.50) showed results consistent with the main linear model. Female sex (*β* = − 1.75, *p* < 0.001), BED (*β* = 2.27, *p* < 0.001), current psychiatric treatment (*β* = 0.70, *p* < 0.001), and older age (*β* = 0.10, *p* < 0.001) continued to show a significant relationship with BMI. BDD was not statistically significant (*β* = − 0.34, *p* = 0.288), though the direction of the association was preserved. The Y-BOCS total score was non-significant (*β* = − 0.022, *p* = 0.159), consistent with the primary analysis.

In the sensitivity analysis excluding individuals with eating disorders (BED, bulimia nervosa, and anorexia nervosa; *n* = 871), the pattern of relationships remained unchanged. Female sex (*β* = − 1.54, *p* < 0.001), current psychiatric treatment (*β* = 0.90, *p* < 0.001), older age (*β* = 0.088, *p* < 0.001), and BDD (*β* = − 0.90, *p* = 0.007) continued to show a significant link with BMI. Sex-stratified analyses showed a consistent pattern across males and females. In males, BED (*β* = 3.41, *p* < 0.001), current psychiatric treatment (*β* = 0.96, *p* = 0.010), and age (*β* = 0.08, *p* < 0.001) were significantly associated with BMI. In females, BED (*β* = 2.85, *p* < 0.001), current psychiatric treatment (*β* = 0.97, *p* = 0.002), and age (*β* = 0.09, *p* < 0.001) remained significant. No significant interaction was observed between sex and BED (*β* = − 0.55, *p* = 0.49), indicating no evidence of effect modification.

## Discussion

### Key findings

This study aimed to investigate associations between BMI and sociodemographic and clinical variables in a large OCD sample (*n* = 947) from seven OCD clinics in Brazil. Participants had a mean BMI in the normal weight range (24.55 kg/m^2^). According to WHO criteria, 5.5% of participants were classified as underweight, 58% as normal weight, 25% as overweight, and 11.5% as obese [28].

In adjusted models, increased BMI remained independently associated with older age, a small effect per year (*β* = 0.09 kg/m^2^) that may become cumulatively meaningful across the adult lifespan, as well as with male sex and current psychiatric treatment, both modest effects. The strongest predictor in the multivariate model was BED, a large effect corresponding to nearly a full BMI category shift (e.g., from normal weight to overweight), reinforcing the robustness of this link. Conversely, BDD was related to lower BMI, a small effect of comparable magnitude in the opposite direction. This association was not replicated in the median-based quantile regression, suggesting that the effect is concentrated among individuals with extreme (particularly low) BMI values rather than representing a generalized shift across the sample. This tail-specific pattern warrants cautious interpretation. No independent relationship emerged between BMI and OCD symptom severity. Taken together, the consistency of these results across sensitivity analyses reinforces the robustness of the central findings: BMI variation in this OCD sample appears more closely tied to demographic, comorbid (particularly BED), and treatment-related factors than to OCD severity itself.

### Comparison with previous literature and interpretation of findings

A lower prevalence of overweight (25% versus 48.1%) and obesity (11.5% versus 15%) is observed compared with the general Brazilian population during the same timeframe. Results should be interpreted within the temporal context of data collection (2003–2009), as obesity prevalence in Brazil has increased over time [29]. These comparisons, for exploratory use only, used data from the 2010 *Surveillance of Risk and Protective Factors for Chronic Diseases by Telephone Survey* (VIGITEL). The assessment involved 54,339 participants, one of Brazil’s most comprehensive sources on chronic disease risk factors [30]. Both our study and the survey used self-reported weight and height from adults.

Patterns in both datasets showed that men generally had higher BMI, with overweight and obesity more common among them. In comparison, women more often had lower BMI, indicating a consistent pattern despite no formal comparison. It is important to note that our sample was drawn from patients attending OCD specialty centers in cities across different regions of Brazil [22]. BMI outcomes did not differ significantly between centers, suggesting that regional variability was not related to the results. In contrast, the VIGITEL survey includes data from adults living in all Brazilian state capitals and the Federal District, providing a broader representation of the general population [30].

Previous studies have hypothesized a lower prevalence of overweight and obesity among individuals with OCD [9, 10]. A possible interpretation for the lower BMI in OCD subjects is that individuals with higher levels of perfectionism and obsessive symptoms appear to show a greater prevalence of disordered eating behaviors [21, 31]. Patients with OCD may have more rigid eating behaviors and a stronger tendency toward orthorexia [19]. Brierley et al. (2021) found that patients with severe OCD may have heightened obsessive and compulsive symptoms during meals [20]. These hypotheses remain speculative and cannot be tested within the present cross-sectional design.

BMI comparisons between individuals with OCD and the general population have been addressed in prior research. While Hennighausen et al. (1999) examined a sample of adolescents with more severe psychiatric conditions and the presence of eating disorders, Abramovitch et al. (2019) focused on an adult sample without eating disorders [9, 32]. Notably, both studies reached similar conclusions: individuals with OCD tend to have lower BMI compared to the general population. However, the second one found this connection only among those with a pure OCD phenotype, that is, without comorbid depression, suggesting that major depressive disorder was a key mediating factor in weight gain within this population [9]. The current study did not observe a link between BMI and comorbid depression. Of note, our study was observational, not comparative. The discrepancy may reflect differences in population characteristics, medication status, cultural context, or the interplay of other comorbidities, such as BED and BDD, that were associated with BMI in the current study but not considered in Abramovitch’s study.

A positive link between age and BMI in OCD patients was observed, with BMI increasing on average by 0.09 kg/m^2^ per year, suggesting a small but cumulative association over time. This is consistent with the cohort study by Isomura et al., which assessed the risk of metabolic and cardiovascular complications, including obesity in individuals with OCD, and observed a progressive increase in obesity with aging [8]. According to the cohort, the use of selective serotonin reuptake inhibitors (SSRIs), whether or not they were combined with antipsychotics, was associated with a reduced cardiovascular risk, particularly at moderate to high doses and with treatment durations longer than one year [8]. Although the present study did not assess the impact of psychotropic medication use on metabolic complications, we found that patients currently undergoing psychiatric treatment, including those using psychopharmacological agents, were characterized by higher BMI levels. However, this should be interpreted cautiously, as current psychiatric treatment likely reflects a combination of illness burden and exposure to psychotropic medications rather than a causal effect of treatment itself.

A positive correlation was found between BMI and BED, which is consistent with prior literature, since this condition often co-occurs with comorbid obesity and involves frequent episodes of excessive food intake accompanied by a perceived inability to regulate eating behavior [33, 34].

On the other hand, BDD showed a significant negative association with BMI in the main linear regression model (although not retained in the median quantile regression, with direction preserved), which is noteworthy, given that body weight is not typically a core element in the psychopathology of BDD. Unlike bulimia and anorexia, where focus is on body weight or shape, individuals with BDD worry about perceived flaws, often facial features, or feeling unattractive or deformed [35]. Despite these differences, comorbidity between BDD and eating disorders is possible, with studies reporting co-occurrence rates ranging from 4 to 15% in adults and 10% to 17% in adolescents [35]. Anorexia and bulimia nervosa did not show statistically significant associations in the bivariate analysis. Nevertheless, given their low frequency in our sample, these results should be interpreted with caution, as the analyses were likely underpowered to detect small or moderate effects. Even so, we cannot rule out that subthreshold body image problems may be connected to lower BMI in our sample. BDD, by contrast, was present in 106 individuals, providing greater robustness for the analysis.

A speculative interpretation for the negative correlation between BMI and BDD observed in this study may lie in the drive for thinness commonly seen in individuals with BDD. As demonstrated by Cerea et al. (2022), in individuals seeking cosmetic procedures who exhibited BDD symptoms, these symptoms were predicted by the severity of “not just right experiences” (NJREs), age, and drive for thinness [18]. This hypothesis requires further investigation to elucidate the mechanisms underlying this association in individuals with OCD.

The multivariate model did not indicate a link between BMI and OCD symptom severity. This lack of association may reflect overlapping variance with other variables, such as current psychiatric treatment, which may involve the use of medications that contribute to weight gain, comorbid BDD, or BED. Our findings align with those of Abramovitch et al., as their study also failed to identify an independent relation with OCD severity and BMI in the multivariate model. However, as previously mentioned, their results differ from ours in that comorbid depression emerged as the only significant predictor of BMI among patients with OCD [9].

Although our study did not find a correlation linking symptom severity to BMI variation, existing literature shows an association of lower BMI with increased risk and prevalence of OCD. Genetic research has also noted an inverse relationship between BMI and susceptibility to OCD [5, 11]. Risk alleles for OCD have been found to be negatively related to BMI, reinforcing the hypothesis of a shared genetic predisposition between OCD and lower body weight, one that is not solely explained by behavioral, cultural, or pharmacological factors [11]. Subramaniam et al. also found that being underweight was associated with both lifetime and 12-month OCD diagnoses. However, this finding may partially reflect an overlap between anorexia nervosa and OCD among underweight individuals in their sample [10].

It is important to note that BMI alone may not be a sufficient or reliable indicator of physical health in individuals with OCD. While BMI remains a practical and widely used screening tool in public health and clinical settings, it provides only a crude estimate of adiposity. It fails to capture important aspects such as body composition, fat distribution, functional health, and metabolic risk. Metabolic measures are now considered essential for the clinical diagnosis of obesity and for assessing cardiovascular complications [36]. In this context, incorporating complementary anthropometric, metabolic, and performance-based metrics is essential for clinical evaluations [37]. These considerations are particularly relevant in subjects with psychiatric diagnoses, where behavioral patterns, neurobiological factors, and comorbid conditions may relate to body weight, body composition, and overall physical health [38]. Therefore, a multidimensional approach to health assessment is warranted to understand the complex relationship between somatic and psychiatric health in individuals with OCD, particularly in future research.

### Strengths and limitations

This study has several important strengths. It is based on a large multicenter sample, including participants recruited from seven specialized OCD centers across different regions of Brazil, thereby enhancing statistical power, representativeness, and external validity within clinical populations. Diagnoses and clinical assessments were established using standardized and validated instruments, ensuring reliability and comparability across sites. In addition, the robustness of the findings was supported by multiple complementary and sensitivity analyses, including median-quantile regression, sex-stratified models, and a comparison between ordinary linear and mixed-effects models, which yielded consistent results across different modeling approaches and assumptions.

Several limitations should be acknowledged. The sample was drawn from specialized OCD centers, representing a treatment-seeking population with more severe illness. This introduces potential referral and selection biases. Using self-reported weight and height to calculate BMI may introduce information bias, as individuals tend to underestimate weight and overestimate height [39], which can lead to systematic underestimation of BMI and attenuation of observed associations. This may be particularly relevant in individuals with body-image-related psychopathology, such as BDD or eating disorders, where perceptual distortion may further affect reporting accuracy. Nevertheless, self-reported measures are widely used in epidemiological research due to their feasibility, and previous studies, including some in patients with BED, anorexia nervosa, bulimia nervosa, and severe mental illness, have shown that self-reported weight correlates reasonably well with objective measurements [40–46].

Another limitation is the lack of objective metabolic measures and the absence of key behavioral and pharmacological determinants of BMI. Variables such as diet, physical activity, smoking, and alcohol use were not available in the dataset, and detailed longitudinal data on pharmacological history, including medication type, dose, and duration of exposure, were also lacking. This is particularly pertinent given that SSRIs and antipsychotics are linked to weight gain and may confound the observed associations between OCD and BMI, especially by contributing to higher BMI among individuals receiving treatment [15, 16]. The absence of these variables likely introduces residual confounding, particularly for predictors such as current psychiatric treatment and comorbidities, and is consistent with the modest proportion of variance explained by our multivariable model (adjusted *R*^2^ = 0.16), suggesting that additional unmeasured factors contribute meaningfully to BMI variability beyond the variables included.

Additionally, the period of data collection may restrict the generalizability of the findings to contemporary clinical contexts. These findings should be interpreted in light of temporal changes in obesity epidemiology. National surveys show a substantial increase in overweight and obesity among Brazilian adults over time, with obesity rising from 11.8% in 2006 to 21.5% in 2020 and overweight rising from 42.6% to 57.5% [29]. Importantly, since all data were collected within the same time window (2003–2009), the within-sample associations between BMI and clinical/sociodemographic variables are not affected by subsequent shifts in population-level obesity prevalence.

In conclusion, in this large clinical sample of individuals with OCD, BMI was not independently associated with OCD severity in the multivariable model. Instead, BMI was significantly associated with age, sex, BED, and current psychiatric treatment status, while an inverse association with BDD was observed in the main model but was not consistent across sensitivity analyses. To our knowledge, this is one of the first studies to examine these associations in a South American population. These findings suggest that BMI variation in OCD appears more closely related to demographic and clinical factors than to symptom severity itself. However, given potential residual confounding and the absence of detailed metabolic and behavioral data, these associations should be interpreted cautiously. Longitudinal studies in low- and middle-income countries are needed to better understand the temporal dynamics between BMI and OCD-related variables. Future research should also incorporate more comprehensive measures of metabolic health and lifestyle factors and investigate the underlying mechanisms of these associations.

## What is already known on this subject?

Although many psychiatric disorders are linked to higher BMI and cardiovascular risk, findings in OCD are inconsistent. Some studies report increased metabolic risk among individuals with OCD, while more recent evidence suggests an inverse correlation between BMI and OCD risk, including a negative genetic correlation. Patients with OCD, especially those without depressive comorbidity, may present lower BMI or a higher prevalence of underweight [5–10].

## What this study adds?

This is the first large multicenter study from a low- and middle-income country to examine the relationship between BMI and OCD. The findings indicate that BMI was not associated with OCD symptom severity, suggesting that body weight variation in this population may be more correlated with demographic and clinical factors than with the severity of obsessive–compulsive symptoms themselves. Additionally, to our knowledge, this is one of the first studies to report an inverse association between BDD and BMI among individuals with OCD, a finding not previously reported in the literature.

## Data Availability

The datasets generated during and/or analyzed during the current study are available from the corresponding author on reasonable request.
